# “*No one was coming to save us*”: an interpretative phenomenological analysis exploring the experience of parents supporting their autistic daughter through anorexia nervosa

**DOI:** 10.1186/s40337-025-01420-w

**Published:** 2025-11-13

**Authors:** Laura Pettitt, Rose-Marie Satherley, Lucy Hale

**Affiliations:** 1 University of Surrey, Stag Hill, Guildford, GU2 7XH UK; 2 King’s College London, Strand, London, WC2R 2LS UK

**Keywords:** Anorexia nervosa, Autism spectrum conditions, Family-based treatment, Outpatient, Carers

## Abstract

**Background:**

Caring for someone with anorexia nervosa is associated with high levels of carer burden and burnout, however, there is a lack of research into caring for individuals who have anorexia nervosa and are also autistic, despite high levels of co-occurrence. This study aimed to offer an in-depth exploration of experiences for this group of caregivers.

**Methods:**

Semi-structured interviews were conducted with six parents with an autistic daughter who had experienced anorexia nervosa. Data was analysed using Interpretative Phenomenological Analysis which enabled in-depth exploration of carers’ lived experience.

**Results:**

Three themes and seven sub-themes were identified. These explored the experience of eating disorders services as largely unprepared to work with dual diagnosis; the impact of their daughter being autistic on carers’ experience of anorexia nervosa treatment and recovery, with variation depending on several factors; the journey of parenting through anorexia nervosa, and changes to parenting as a result.

**Conclusions:**

This adds to our understanding of the lived experience of this group of carers, highlighting a need for early detection of autism spectrum conditions, enhanced staff understanding of autism spectrum conditions, tailored treatment, and specific carer support for this group.

## Plain language summary

Parents of a young person who is autistic and also has anorexia nervosa face unique challenges because of the needs that come with both conditions. The study looked at the experience of parents supporting their autistic child through Anorexia Nervosa. Parents told us that eating disorders services did not feel prepared to meet their child’s needs. In response to this, many felt that they had to change or even stop treatments because they didn’t work well for their child. Parents also spoke about the impact this had on their own wellbeing and their family. They described how they often had to step into unexpected roles, such as advocating for their child with professionals. The findings suggest that treatment for anorexia nervosa may be more helpful if it included adaptations for autistic people, and that providing support for carers could make a big difference for both parents and their children.

## Introduction

Research currently estimates that 29–40% of individuals with anorexia nervosa (AN) exhibit elevated autistic traits, and autistic individuals are five times more likely to experience disordered eating compared to neurotypical peers [12, 22, 53, 55]. Shared features, such as sensory differences, food selectivity, rigidity, cognitive inflexibility, and interpersonal difficulties, suggest an interplay between the two conditions [4, 45, 62]. Both AN and ASC have been observed to have neuropsychological parallels, including a preference for logical, detailed, and pattern-based processing rather than global processing (often referred to as 'weak central coherence'), and differences in social processing [44], raising questions about whether these conditions share underlying cognitive mechanisms. It is important to note that social differences between autistic and non-autistic people often lead to the misunderstanding and framing of autistic people as having social and empathy 'deficits', rather than as mutual communication differences that occur due to diverse neurocognitive profile [13, 39].

Despite growing recognition of this overlap, the nature of the underlying dynamics remain unclear. Individuals with AN frequently score higher on autistic trait self-report measures [3], yet most do not meet full diagnostic criteria for autism spectrum conditions (ASC) when assessed formally [63]. This discrepancy has fuelled debate about whether these traits reflect a stable neurodevelopmental profile or are state-dependent, emerging as a consequence of starvation [71]. While some findings support the latter hypothesis, others have shown that social and cognitive traits associated with ASC persist post-recovery, suggesting more enduring autistic characteristics [7, 23]. Longitudinal research has suggested that young people showing higher levels of social communication differences associated with ASC were more likely to develop disordered eating behaviours in adolescence, suggesting that these autistic traits may pre-date the onset of an eating disorder [60]. Moreover, autistic populations consistently report elevated AN symptomatology even after nutritional rehabilitation, underscoring the potential bidirectional nature of this relationship [70].

Autistic neurocognitive features and sensory differences may also be misinterpreted as solely eating disorder-related, a phenomenon known as diagnostic overshadowing, particularly for individuals who have not yet received an autism diagnosis [11, 12, 16, 46]. Such misinterpretation can contribute to delayed or missed autism diagnoses and may result in distressing treatment experiences and/or non-response [12, 16]. To address this, research and treatments may need to be co-designed and tailored to autistic populations in order to improve outcomes [8, 12, 31].

Autism diagnosis can differ according to gender, with cisgender females, as well as transgender and non-binary individuals, often receiving a later diagnosis than cisgender males [20, 33, 37, 46, 47]. Delays, compounded by barriers such as long wait times, high costs, and limited service accessibility [30, 35, 72], can contribute to diagnostic overshadowing and treatment gaps in eating disorder care [49].

AN has the highest mortality rate of any psychiatric disorder [15], and outcomes are notably poorer for autistic individuals, who often experience longer illness durations, greater severity of symptoms, and increased treatment non-response [6, 42, 52, 61]. Standard treatments may be poorly suited to this population, as they often fail to account for sensory sensitivities, communication differences, and cognitive rigidity [2, 5, 18, 54]. In response, adapted approaches such as the Pathway for Eating disorders and Autism Developed from Clinical Experience (PEACE) pathway have been developed to tailor eating disorder treatment to autistic adults with AN, incorporating sensory screenings, modified meal plans, and ASC-informed psychoeducation [64].

Given the common onset of AN during adolescence [41], caregiving responsibilities often fall to families, who report high levels of distress and burnout when supporting young people (YP) who are either autistic or have AN [2, 10, 19, 68]. In the UK, the National Institute for Health and Care Excellence (NICE)-guidelines recommended treatment for young people with anorexia nervosa is anorexia nervosa-focused family therapy [34], which may be described as family-based treatment (FBT), FT-AN or the Maudsley Approach [50]. These approaches to AN treatment involving the family taking control of the young person’s eating until weight restoration occurs [56], meaning that family members are often heavily involved in the YP’s care and treatment [66].

Yet, the experiences of caregivers supporting autistic YP with co-occurring AN remain underexplored [1, 17], despite some evidence of heightened caregiver burden in both groups. Focusing on caregiver experience and their unique challenges, such as the impact on family life, engagement with services, and methods of adapting treatment, may enhance understanding of why treatment is less effective, and provide insight into how families should be supported as a system. Existing qualitative studies have began to explore parental experience of caring for an autistic individual with AN (e.g., [1, 2, 5, 9]). However, in these studies, parents are typically one of several participant groups, meaning that parental voices were not the focus of analysis. The present study builds upon this literature by using interpretative phenomenological analysis (IPA) to conduct an in-depth, idiographic exploration focused exclusively on parents’ experiences. This approach provides a richer understanding of the identity shifts, systemic challenges, and unmet support needs that parents face when caring for an autistic young person with AN, to inform the development of more inclusive, system-wide support for families.

## Methods

This study used Interpretative Phenomenological Analysis (IPA), a qualitative methodology that aims to explore in depth how individuals make sense of their lived experiences [59]. IPA is based on phenomenology, hermeneutics and idiography, and is described as double hermeneutic,the researcher’s own position is considered, as they try to make sense of how the participant is understanding their own experience [59]. It is therefore a reflexive process which leads to a co-constructivist approach. By considering the influence of the researcher on the data, new meanings can be generated [58]. For the researcher’s reflexivity statement, please see Appendix B.

All participants provided verbal and written informed consent. Ethical approval was obtained from the Health Research Authority (HRA) following approval from the East of Scotland Research Ethics Service (LR/22/ES/0043).

### Participants

The present study forms part of a broader qualitative project involving 13 caregivers of autistic YP with a diagnosis of AN who had received at least one session of Family-Based Therapy (FBT) within the past 9 months. From the broader sample, six parents were purposively selected for their rich and reflective accounts, offering both shared and divergent perspectives consistent with IPA’s idiographic focus [59]. While there were some demographic differences between participants, for example, one participant was a father, differences in treatment types and recovery status, Larkin et al. [28] describes how differing perspectives can demonstrate an underlying structure of experience or enable exploration of how different stances relate to each other.

All participants were parents of autistic daughters. Five participants were mothers, and one was a father. The mean participant age was 53 years (SD = 3.44), and their daughters’ mean age was 17.5 years (SD = 3.15). The minimum age of participants was 18 years. Regarding the age of the young person the participant was caring for, they were required to be under the age of 18 or could be older if they had had at least one session of FBT in the prior 12 months. This was because some services may offer FBT to transition age adults up to 25 years old [14]. Thus, while most parents were caring for adolescents, a proportion were supporting young adults who were offered a family-based approach beyond the age of 18. Prior to transcription, all participants were allocated a pseudonym by the lead researcher to ensure anonymity. The pseudonyms were selected to reflect participants’ gender but had no other identifying features.

Participants’ daughters had experienced a variety of treatment types and were at different stages of treatment and recovery. For example, some parents’ daughters had only received outpatient treatment, others requiring inpatient admissions, general hospital admissions or day unit care. All participants’ daughters had been formally diagnosed as autistic, though there was variation in timing of diagnosis. All participants were white British. For additional demographic details, see Table 1.

**Table 1:** Participant demographics

Pseudonym	Age	Gender	Young person’s age	Young person’s gender	Age of ASC diagnosis	Age of AN diagnosis	Do they consider their daughter to have recovered from AN?
Helen	50	Female	19	Female	18	13	No
Linda	57	Female	19	Female	15/16	18	No
Mel	56	Female	14	Female	14	14	No
Lindsay	52	Female	14	Female	10	14	Yes
Kevin	56	Male	22	Female	22	19/20	No
Ros	49	Female	17	Female	15	15	Yes

Participant number was limited to six based on recommendations by Turpin et al. [67] for sample sizes for qualitative doctoral research. Additionally, Vasileiou et al. [69] suggested that for IPA, sample sizes under ten enabled in-depth analysis.

### Procedures

Participants were mainly recruited via targeted social media advertisements, though two were recruited through NHS eating disorders services (EDS). Recruitment took place between January and June 2023. Upon providing consent, interviews were scheduled based on participant preference, via telephone, Microsoft Teams, or in person.

Interviews lasted approximately 90 min and were guided by a semi-structured topic schedule. The topic guide was developed by the lead researcher (LP) and amended in collaboration with a service user and carer representative committee (see Appendix A). Participants received a £10 voucher as a token of participation.

### Analysis

Interpretative Phenomenological Analysis (IPA) was chosen for its suitability in exploring under-researched and highly emotive topics [48]. By focussing on the details of lived experience and the meanings individuals ascribe to their experiences [57], IPA is particularly well-suited to understanding the complex emotional, relational, and systemic challenges faced by parents supporting an autistic young person with AN.

Interviews were audio-recorded, transcribed verbatim, and analysed using IPA, following the procedures outlined by Larkin et al. [27]. Each transcript was read multiple times, and personal experiential statements (PES) were extracted to facilitate a deep understanding of each participant’s meaning-making. These PES were examined across cases to identify points of convergence and divergence, leading to the development of themes and sub-themes grounded in the data.

Analytic rigor was enhanced through regular supervisory discussions, a reflective log and peer debriefing. The lead researcher was a trainee clinical psychologist (LP) with prior clinical experience in EDS. This background informed the research focus and interview approach while necessitating continuous reflection on potential assumptions, see Appendix B for researcher’s positioning statement.

To ensure analytic quality, the four principles of excellence in IPA proposed by Nizza et al. [43] were explicitly followed: (1) crafting an unfolding narrative, (2) presenting a vigorous experiential account, (3) maintaining close attention to participants’ words and meanings, and (4) identifying both shared and divergent experiences. These principles were referred to continuously throughout the analytic process and discussed in supervision to refine interpretation and enhance validity.

## Results

Three superordinate themes and seven subthemes were developed. These are summarised in Table 2.

**Table 2:** Superordinate and subthemes

Superordinate theme	Subthemes
Parenting in the absence of professional containment: “*No one was coming to save us”*	Where does the responsibility lie for getting my daughter better?
	As a parent I had to “*fight*” for my autistic daughter
A journey of understanding my daughter’s dual diagnosis	*“Taking away choice and not collaborating with her was never going to work”*: we couldn’t follow typical AN treatment
	*“Now we know about the autism diagnosis, I get it”*
The impact of parenting my daughter through AN	*“We've learnt to parent in a different way”*
	“*It dominates absolutely everything*”: The overwhelming impact on family life
	*“I just thought ‘I can't do this anymore and I don’t want to be here’*”: Effects on parental wellbeing

### Parenting in the absence of professional containment: “No one was coming to save us”

Parents’ narratives highlighted a growing realisation that services were unequipped to support their autistic child with AN. This created a perceived vacuum in care, compelling parents to assume roles typically held by professionals (e.g. therapist, case manager, advocate), often perceiving this to occur without support or guidance. There was a collective sense of abandonment, frustration, and ultimately, self-reliant adaptation in the face of systemic inadequacy.

#### Where does the responsibility lie for getting my daughter better?

Parents began their journey believing EDS would lead treatment. However, they soon encountered a system they perceived as inconsistent, poorly informed, and ill-prepared for the complexities of the needs of a young person being both autistic and having AN. Helen captured this disillusionment: “*all professionals are too rubbish at their jobs… they should be doing the job that they're being paid for”.* Her frustration was evident, grouping *“all”* professionals suggesting deep erosion of trust and a sense of betrayal. The emotional tone conveyed not only anger but a collapse in confidence in the very system that was meant to help.

Others described the difficult transition from passive recipient of care to primary carer. Linda, reflecting on the intensity of her involvement, said: *“we've come to you for treatment and you're… telling me I've got to do it. I'm not even trained.”* Initially resistant, she ultimately reframed her role as essential: “*There’s no way anyone else in the world is going to be doing this except me… it has to be family-based.”*

This evolution in role reflects a broader identity shift: parents came to view themselves as experts-by-experience, embodying a care model that they felt services could not provide. However, this was born of necessity rather than choice.

The phrase repeated by multiple parents, “*no one is coming to save us,”* spoke to the existential weight of this realisation. Ros, reflecting on her daughter’s discharge from inpatient care, stated: “*There was no feeling that services were going to… help to get my child better… I very quickly realised that it was really down to us.”* Similarly, Lindsay shared: “*We sat down as a family and said, ‘nobody’s coming to save us. We have to do this ourselves.*’”.

This marked a turning point for many parents: an internal shift from expecting external rescue to accepting full responsibility. The absence of systemic containment forced families to become both the scaffolding and the safety net.

#### I had to “fight” for my autistic daughter

The sense of abandonment was compounded by the need to advocate, often forcefully, for ASC-informed care. Parents used language of combat and persistence, describing repeated efforts to have their daughter’s autistic needs recognised and respected. Helen described feeling that autistic people *“don’t even have a voice.”* The perception that her daughter’s voice was actively silenced reflects both protective instinct and moral outrage. The fight was not just for access but for recognition of personhood. Ros’s metaphor of advocacy as disruption was particularly vivid: *“I was banging the drum the whole time… it was down to me as the parent to, to bang the autistic lens drum of treatment”.* According to Ros, without this advocacy, her daughter could have been overlooked in critical treatment decisions. The image of the drum suggested that this advocation was perceived as a disruptive to the usual course of treatment.

This familial knowledge served as a foundation for resisting treatments perceived as unsuitable or harmful. Ros went so far as to decline care altogether: *“she basically refused to engage with any NHS professional … Again, through the autistic lens I was like, ‘that's absolutely fine’, I cut everything back, I was like, ‘no, she's not doing this”.*

Parents did not just observe shortcomings in care, they actively reshaped it, often in opposition to professional advice. Linda expressed the anticipatory vigilance required in each new professional encounter: *“The first thing I say to everybody is, ‘have you got any experience with autism?’… if they don’t, I have to put them in the picture.”*

These accounts reveal that parents felt forced to occupy the space left vacant by a system not equipped to support dual diagnosis. This dual role of both carer and advocate was emotionally costly but became a perceived necessity for safeguarding their daughter’s well-being.

### A journey of understanding my daughter’s dual diagnosis

Participants collectively acknowledged the impact of their daughter being autistic on their experience of AN. For some, they could anticipate how the conditions would interact, while others gained this knowledge during AN treatment, shaping parental narratives. The experiences reflected shifts in understanding, reframing of responsibility and blame, and the relational empathy which developed as parents’ narratives were reshaped through retrospective meaning making.

#### “Taking away choice and not collaborating with her was never going to work”: we couldn’t follow typical AN treatment

Parents experienced aspects of AN treatment as in direct conflict with their daughter’s autistic traits, and a hinderance to their daughter’s ability to engage and thus recover. Parents described their initial reaction to conventional treatments as “*overwhelming”* (Mel) and *“horrendous”* (Linda).

Parents appeared to derive a sense of power by attributing the ineffectiveness of conventional AN treatments to their incompatibility with autistic traits. A collective identity seemed prevalent across parents’ narratives, alluding to the “*autistic experience”* (Lindsay), refraining from portraying experiences as exclusive to their daughter, while also acknowledging that *“autism is not the same in each person”* (Linda). Ros described how *“I'd already read a lot and I knew that FBT wasn't a good fit for autistic people*”, giving her confidence to challenge the treatment offered. This knowledge empowered parents to scrutinise treatment approaches, and request or make modifications to align with their daughter’s needs.

Parents felt that their daughter’s needs as an autistic person necessitated they took on a prominent role within treatment than other carers of YP with AN. Mel described needing to speak on her daughter’s behalf in sessions, “*she wasn't treatable, she wouldn’t engage*”. Linda considered the complexities of her daughter being autistic as evidence of the importance of parental involvement in treatment: “*this is why it's got to be the primary carer, because they are the ones that know the person and you're not dealing with textbook when you've got autism in the mix”*. This highlighted an idea that clinicians were used to working with a singular approach, and that parents needed to advocate for their autistic daughters to request individualised treatment.

Rather than just being unhelpful, Ros felt that treatment would actively harm her daughter: *“dictating and taking away choice and not collaborating with her was never going to work. It was just going to, uh, entrench her patterns of behaviour”.* These parents’ understanding of their daughter’s needs as an autistic person gave them the confidence to challenge conventional treatment offers before attempting to implement them.

Parents unaware that their daughter was autistic prior to her AN seemed less confident in challenging treatment offers, taking a passive role of consumer of healthcare, powerless to question treatment. This contributed to distress, as Mel described the backlash from her daughter: *“she attacked me and grabbed hold of my hair um and wouldn't let go and she was kicking me … wasn’t the daughter I knew”.* The treatment was felt to be so inappropriate that it made Mel’s daughter unrecognisable.

Similarly, inpatient care was considered poorly suited to autistic people with AN. For Helen’s daughter, her expectations of healthcare to get their daughter better were not only not met, but instead the system was failing her, and making her more unwell. Helen described the impacts of an inpatient admission: “s*he was discharged … because she was so distressed, getting still even worse. She's come out even worse. It's horrific, the state she's in”.*

Realising that conventional treatments would be ineffective, led to parents stepping into advocating roles or making adaptations to treatment. For Lindsay, this meant changing the “rules” which they felt had been imposed by EDS: *“we did do a bit, a fair bit of rule bending … essential for her [daughter] to get better”.* For others, this meant rejecting conventional treatment entirely, as Ros explained: *“basically rip up the meal plan, which is what we did. Felt very uncomfortable, but … then she responded well”.* Ros’ discomfort in rejecting treatment was clear, suggesting a cognitive dissonance experienced by these parents.

Some sought peer support to connect with other carers to help process their daughter’s AN and their caring experience. However, difficulties in relating to the experiences shared by friends or in formal support groups made this experience isolating. It was felt that their experience of AN was different to that of other carers due to dual diagnosis, as Kevin explained: “*I never really found that the way other people did things were relevant. Maybe it’s ‘cause Lucy's autistic and their kids weren’t … but I was just thinking … ‘that's not my experience’ … ‘that's not the way it is for Lucy’”*.

Parents’ understanding and acceptance of their daughter’s autistic identity developed through their experience of AN, as their insight into their daughter’s needs grew. It seemed that parents realised that their deep understanding of their daughters was not accounted for by typical AN treatment, despite being crucial for their daughters’ recovery. This led to their understanding of treatments’ shortfalls deepening over time, with blame shifting from themselves and EDS, onto the role of ASC in making conventional treatments feel irrelevant and needing adaptation.

#### “Now we know about the autism diagnosis, I get it”

Parents highlighted how their relationship with and understanding of their daughter’s dual diagnosis developed over time. It appeared that parents developed relational empathy in now understanding their daughter's experiences through an autistic lens, as Helen described: *“we were looking at old photos and all the birthday parties she’s got her hands over her ears. Never noticed, every single photo, you know?*”. It seemed that the diagnosis helped them to retrospectively ascribe meaning to their daughter’s life experiences, deepening their understanding of her and connection to her.

For those whose daughter was diagnosed as autistic prior to developing AN, this understanding of their daughter’s needs as an autistic person helped parents to anticipate and avoid challenges, as Linda described: *“if I put too much pressure on her, she would then start to get stressed, or feel she was being pushed and manipulated and she'd lost her control”.* It seemed that parents were on a journey of understanding their daughter’s identity, as Ros described her understanding and acceptance of her daughter: *“I always say about her, her, her beautiful autistic brain”*.

For those whose daughter was diagnosed as autistic during AN treatment, this offered an explanation for the ineffectiveness of AN treatment, seeming to shift parents’ self-blame and reframe it as a systemic issue. Mel described how *“I tried so hard, so, so hard to … get her to open up and to tell me what was going on, what was wrong, and she never could … now we know about the autism diagnosis, I get it”*, suggesting that parents felt able to connect better with their daughter with this newfound deeper understanding of her.

While ASC diagnosis may have led to personal clarity, this did not always translate into professional support. Helen had expected clinicians to support her daughter’s needs with a new diagnosis but instead experienced this as being used as an excuse to not provide care-* “that's fine 'cause you're autistic’. So, it's fine not to eat because she's autistic … because it would overwhelm her to eat, so best not get her overwhelmed”.*

### The impact of parenting my daughter through AN

Parents reflected on the impact of caring for their child through AN, with effects on their identity, relationship with their daughter, and their own wellbeing. Parents’ narratives told of the challenging experiences they faced, and the trauma this caused them. The theme captures the parents’ embodied experience, and the exhaustion and fear which they experienced due to the evolution in their parenting role.

#### “We've learnt to parent in a different way”

Parents’ narratives explored the impact of dual diagnosis on their parenting techniques, describing how the new challenges posed by AN forced them to reconsider and adapt their parenting, and the adaptations which occurred in response to the repeat crises which their daughter being autistic and having AN brought to their family. Mel reflected on this: “*I think we've all been on a journey and, you know, we've learnt to parent in a different way… we do it in a very different way now”.*

The role parents had expected to occupy had shifted, from parent, to carer, to advocate, and while this seemed to come as a surprise for parents, their narratives were not recollections of resentment but encompassed the shock which they felt due to this change in role. Their narratives described a lack of preparation for this, as Mel described: *“like a baptism of fire, I'd been thrown into becoming a full-time carer,”,* with Linda similarly describing having to bathe her adult daughter.

This evolution in role was self-directed; parents described no guidance from EDS, as Ros described: *“I had to really do a lot of work myself and really question, educate myself and really change my approach completely to get to where we needed to be”*, seeing adapting her own approach to her daughter as being essential for her daughter’s recovery, and needing to approach parenting in a ‘completely’ different way. This notion was mirrored by Mel, who described: *“that was self-taught. That was, you know, it wasn't really anything that the clinic, any therapy that helped us with that”.* This reinforced the notion that parents, armed with extensive knowledge of their daughter and her unique needs due to being autistic, play an irreplaceable role that professionals were unable to replicate.

#### “It dominates absolutely everything”: the overwhelming impact on family life

Within AN treatment, parents’ perceived responsibility appeared to extend beyond caring for their unwell daughter, as they also considered the relational dynamics and impact on their other children. There seemed a perception that dealing with the strain and trauma of their daughter’s AN was part of their parental role, but that siblings should be shielded from these difficulties.

Kevin’s adult son chose to take on a caring role for his sister while she was unwell with AN, and Kevin described the toll that this took on his wellbeing: *“he was her carer effectively … I know he was sort of traumatised by, by that whole experience”.* similarly, Helen expressed concern for what her other daughters witnessed when Beth was unwell: “*her sisters have had to restrain her from jumping off things … seeing her unconscious when she's collapsing. Yeah, they're very traumatised”.* This graphic description highlights the severity of the experience, and impact on the whole family. Mel shared concerns about the impact of her daughter’s illness on her sister, *“and we worried about Molly as well because all the focus was on Layla”.* There was a feeling that the parents were stretched trying to care for their daughter who was unwell, while also continuing to parent their other children; a role which seemed impossible to manage.

Language used by parents when describing AN differed to how they spoke about ASC. AN was perceived as all-consuming, and dangerous, possibly due to its potentially deadly nature, in contrast to ASC, which parents viewed as a lifelong part of their daughters’ identity, as Lindsay described: *“I can see in Emily's eyes when anorexia is around, it's like, a change in personality, it's a change in how her face looks, it’s a change in the tone in her voice. It's… like a possession”*.

Perceived parental role and responsibility seemed to impact upon language choice- perceiving AN as something which they had a duty to support their daughter to recover from, compared to ASC, which it seemed they felt they needed to help their daughter, and others, to understand and accept. There seemed a notion that they needed to adapt their life to support their daughter’s needs as an autistic person, as Lindsay described *“we have to live differently anyway,”* suggesting an acceptance of ASC.

#### “I just thought ‘I can't do this anymore and I don’t want to be here’”: effects on parental wellbeing

Unsurprisingly, parents described the toll that caring for their daughter through AN took on their own mental health, as they navigated their daughter’s treatment and recovery, while fearful of the threat of her mortality.

Mel described how *“I went on to antidepressants in May last year. Um, because I really wasn’t coping … I started thinking about suicide … because I just thought ‘I can't do this anymore and I don’t want to be here’”.* This highlighted the significance of this caring experience on parents, leading them to develop their own mental health difficulties due to acting as the main source of support for their unwell daughter.

Helen described the all-encompassing impact of the experience, with a feeling that the damage caused to her family was irreversible: *“Oh God, it's like it, it, is dominates absolutely everything. I mean, it's destroyed our lives. Absolutely destroyed our lives”,* and Lindsay’s description supported this notion: *“I would say it’s horrific. I think it's possibly the worst thing a parent can experience”.* Parents’ narratives framed their caring role as their duty, which they had adapted to as it evolved without question. This longitudinal reflection was the first time that parents seemed to fully acknowledge the enormity of the experience, and the extent of the toll it had taken. This highlights how crucial it is for EDS to provide support for carers to help them to manage the demands of their daughter’s AN and treatment, and their own wellbeing.

## Discussion

This study offers new understanding of the lived experience of parents navigating the complex and emotionally charged task of supporting an autistic YP through AN. While the challenges of caring for a YP with an eating disorder are well documented [19, 68], this research highlights a distinctive set of family pressures when a YP is also autistic. This extends prior qualitative research which has documented parental disappointment with ED care [1, 6, 18] by providing an idiographic account oof how parents reconstruct their roles and identities in response to systemic shortfalls.

At the heart of participants’ experiences was a profound breakdown in trust, both in services and professionals. Parents described feeling abandoned by EDS that were perceived as unequipped, untrained, or unwilling to adapt care to meet their child’s autistic needs. Their accounts reflect not just frustration, but an existential realignment: from expecting expert-led recovery, to becoming the architect of their child’s care themselves. This mirrors Ryan and Cole’s [51] findings that parents of autistic youth often act as key advocates in mental health contexts and build upon previous research exploring the experience of autistic individuals with AN, such as Adamson et al. [1], and Kinnaird et al. [25]. Their findings also described parents taking on roles which filled gaps they perceived as being left by services, due to a lack of understanding, or systemic barriers. However, in this study, advocacy was not simply an external role, it became the mode through which parents retained agency. The metaphor of “fighting” and being “left to do it ourselves” captures a transition from disempowerment to reluctant ownership. It appears that parents were highlighting a resistance to learned helplessness [38], whereby they had tried to support their daughter but found this to be ineffective, and felt out of control, but rather than becoming passive, helpless victims, ending up reluctantly shifting their role to support and protect their daughter.

The expectation of support gave way to a perceived moral obligation to act, despite emotional and physical exhaustion. This notion echoes the findings of Babb et al. [5], whereby similarly, parents and autistic women perceived treatments to often be inappropriate for autistic individuals, and described feeling let down by services who offered a generic approach without individualising it. It was particularly striking how parents perceived their actions as necessary not only because of system failure, but because their expertise as parents of autistic children was irreplaceable. This belief reframed the parent as both treatment provider and system corrector, occupying a space that clinicians could not fill. Such positioning, while empowering in some instances, was also isolating and unsustainable. Here, we show that parents not only adapted care but positioned themselves as counterparts to services.

Participants described how their parenting evolved radically and often without professional guidance. The findings highlight the strain and all-encompassing nature of this caring role, echoing the findings of Adamson et al. [1], as the carers shared feelings of disappointment in EDS who let them down, leading to them educating themselves to make adaptations at home to try to support their daughter’s recovery. Similarly in the present study, standard approaches to AN were experienced as either incompatible or actively harmful when applied without ASC-specific adjustments. In response, parents developed new strategies grounded in logic, collaboration, and patience. Tactics not taught but self-constructed through trial, error, and love. This transition represents more than behavioural change,it marks a shift in parental identity. Many parents moved from a normative caregiving model to one of clinical interpreter, strategist, and protector.

For those who only discovered that their daughter was autistic during AN treatment, the process was also bound up in retrospective meaning-making and attempts to reconcile past confusion with a new framework that made sense of their child’s distress, resistance, and needs. As reported in work on late ASC diagnosis [29], this reframing often brought relief but also grief for missed opportunities, harmful interventions, and misunderstood needs. This mirrors the experience of Brede et al.’s [9] sample, as none of their sample received a diagnosis of ASC until after their eating disorder developed. They highlight the importance of earlier ASC detection and adaptations to reduce unnecessary suffering, similarly to the parents within the present study.

ASC provided a lens through which parents made sense of the entire treatment journey. Narrative identity theory posits that individuals’ sense of self develops and evolves depending on context and life experiences [36]. It seems that the caring role led parents to develop a collective identity, and that this was an act of resistance, as this role, initially taken on reluctantly, becoming empowering as imposed narratives were rejected, with new meaning developed. In this way, ASC became a narrative anchor, an explanatory framework which made confusing or traumatic experiences coherent. This interpretive shift allowed parents to re-evaluate both their child’s behaviour and their own emotional responses. Importantly, this narrative reframing extended to how parents made sense of themselves. As services failed to adapt, the act of viewing the child’s needs through an autistic lens became a form of resistance against generic treatment, pathologising language, and being marginalised as “just parents.” It also became a means of identity solidarity, affirming their daughter’s worldview in a system that often dismissed it.

ASC was rarely discussed as deficit. Rather, it was framed as difference: a mode of being that demanded adaptation, not correction. This builds upon findings by Field et al. [18], who highlighted the need for AN treatment to accommodate the needs of autistic individuals. The present study showed how parents themselves develop and enact these adaptations in the absence of professional support to reduce distress. In the present study, many parents described how being autistic shaped their daughter’s emotional landscape, her sensory experiences, and her relationship with control and routine, core components of both identity and AN. When care was misaligned with these aspects, suffering deepened. When aligned, there was room for trust, connection, and healing.

### Clinical implications:

These accounts raise questions about the preparedness of current services to work with autistic YP with AN. While models such as the PEACE pathway [64] offer promising adaptations for adult patients, equivalent frameworks for children and families, remain limited. Given the emotional cost and moral burden placed on parents in this study, the absence of structured, ASC-informed approaches for YP is a clear gap. Parents spoke of the need of earlier ASC screening to enable more appropriate and effective care to begin sooner, and prevent the trauma associated with inappropriate treatment. They described a longing for specific peer support where they could speak to other parents of autistic YP with AN whose stories they could relate to and connect with. There appears to be a gap in current carers’ support provision for carers of a young person who is autistic and has AN to have their own spaces, supporting findings by Adamson et al. [1], and Kinnaird et al. [26], whose research recommends additional peer support and psychoeducation for this group of carers. Parents in the present study speak of a fundamental need for AN treatment to place less demands on their child, to go at a slower pace and to encompass their expertise in their children instead of enforcing blanket rules and a ‘one-size-fits-all’ approach to their children, who were being failed as a result. Adaptations such as those proposed by Loomes & Bryant-Waugh [34] may support clicians who feel unprepared to work with this group [24] as they begin to be adopted by EDS.

Interventions that reduce carer burden have already been shown to improve outcomes in AN [65]. But for autistic YP, carer support must also include recognition of neurodivergent needs, a reframing of the family as part of the therapeutic team, and space for parents’ own identity and wellbeing to be supported.

### Limitations and further research:

While this study offers rich, idiographic insight, the sample was limited in diversity and scale. As IPA involves close analysis of few individuals, the experiences described here are not intended to be representative of all caregivers of children with dual diagnosis. Interpretations are necessarily subjective and therefore, the findings of the present study cannot be extrapolated. Participants within this study were largely homogenous in terms of gender identity, ethnicity, and socioeconomic status, which is pervasive among ED research [21]. All participants’ children were female, and while AN is thought to occur in up to ten times more females than males [70], this limits the breadth of this research. Additionally, only one participant was a father, so the lived experience explored was largely the experience of mothers. Furthermore, eligibility criteria required participants to have received support from an EDS, which may have led to bias within recruitment, as research has suggested many barriers to individuals accessing treatment, and the voices of these individuals are not captured in the research [32].

Caregiver perspectives may have been shaped by service type, illness stage, and timing of autism diagnosis. While such variation enriches IPA insights [28], it limits generalisability of findings but highlights important areas for future research. Future research should widen the breadth of participants to enable exploration of more diverse and representative lived experience, such as exploring the experiences of fathers, carers from racially minoritised communities, and those with differing socioeconomic access to services. Additionally, the impact of tailored interventions, such as carer education on ASC, peer-led support groups, and early ASC screening, should be evaluated longitudinally.

### Conclusion

The experience of parenting an autistic YP through AN is one of adaptation, advocacy, and emotional labour. This study highlights how parents navigate systemic shortfalls, reconstruct their identities in the absence of professional containment, and find coherence through an ASC-informed lens, building upon existing literature with an in-depth exploration of these parents’ lived experience. Their stories reveal a care system in need of radical inclusion—one that recognises not just the needs of autistic patients, but the critical expertise and wellbeing of their caregivers.

## Data Availability

Data from participant interviews is not publicly available to protect anonymity.

## Appendices

### Appendix A

**Interview topic guide- part 1**

**Introduction:** I’d like to understand your experience of [your child/the child you care for]’s anorexia nervosa, and your experience of family-based therapy. To start with we’ll talk about the anorexia more generally, and later we’ll look at family-based therapy specifically.

I know that caring for someone with anorexia can be challenging, and I want to learn about what your experience has been of caring for someone who is both autistic and has anorexia.

1. Let’s start the discussion by thinking about when you first noticed changes to [your child/the young person]’s eating.
  2. Could you talk to me about some of these changes?
  3. How did you initially respond to this?
2. [Your child/the child you care for] has a diagnosis of [autism spectrum disorder (which I’ll refer to as ASC)/ has been identified as having high traits of autism spectrum disorder (which I’ll refer to as ASC)]. ASC presents very differently for each individual. Could you tell me about [your child/ the child you care for’s] ASC?
3. How do you experience [your child/ the child you care for’s] difficulties?
  6. What is it like for you as a caregiver?
  7. What are the main emotions you associate with it?
4. How do you respond to your child’s anorexia?
  9. What about your emotional response? Or particular behaviours? Some people talk about making choices for their child, or helping them to avoid things which make them anxious- are any of these relevant for you?
  10. Can you tell me about a specific example of this?
5. Has this experience changed at different points during their eating disorder?
6. Before service involvement/treatment, could you tell me about how you were managing your child’s eating?
  13. What was happening at mealtimes?
  14. Can you tell me about a specific example?
7. What was your experience of involvement from services on the anorexia and your family life?
  16. What was the impact of this?

**Interview topic guide- part 2**

Thank you for taking part so far- this second part of the interview will look more specifically at your experience of family-based therapy, which I’ll refer to as FBT. Are you okay to continue or would you like a short break before we move on?

17. Could you tell me about your experience of FBT?
  18. How did you access FBT?
  19. How many sessions did you attend?
  20. What happened in these sessions?
18. What were the biggest challenges of FBT for you and your family?
  22. Have you found ways to manage this/overcome this?
  23. Could you please give me a specific example?
19. What were the most helpful parts of FBT for you and your family?
  25. Do you think everyone in your family would agree? Why/why not?
20. How do you think [your child/ the young person you’re caring for]’s ASC has impacted on your experience of FBT?
21. What do you think has been the most helpful thing for the [young person you’re caring for/your child]’s anorexia recovery?
  28. Anything else?
  29. Please could you give me a specific example?
  30. Is there anything that you thought it would be helpful for us to discuss today which we haven’t?

### Appendix B: Researcher reflexivity and positioning

As a research team, we were mindful of how our professional backgrounds and positionalities shaped the study. Collectively, we brought diverse clinical and research experience to the project, which enriched our understanding of the data, but also introduced potential biases that required careful reflection.

The first author had previously worked as an Assistant Psychologist in an Eating Disorders Service, with part of their role involving supporting autistic young people with Anorexia Nervosa and their families. This experience was a key motivation for the project but also meant that they started the study with existing assumptions about treatment and recovery. To address this, they sought to minimise potential bias by asking open questions, being guided by participants’ narratives, and maintaining a reflexive journal throughout the process.

One member of the team was a clinical psychologist specialising in eating disorders, who contributed an in-depth understanding of the clinical context, while another had expertise in qualitative research in psychology, offering valuable insights into methodological rigour and interpretive reflexivity. These different perspectives supported critical discussions during data analysis, helped to identify blind spots, and strengthened the trustworthiness of the findings.

We were aware that disclosing professional backgrounds, particularly those within the NHS, may have shaped participants’ perceptions of us as both researchers and clinicians, potentially influencing what they felt comfortable sharing. To mitigate this, we emphasised our role as researchers, and our aim of amplifying parents’ voices to improve care. As none of us were working directly within Eating Disorder Services at the time of interviews, we hoped that this reassured participants and encouraged open discussion. At times, the first author, who interviewed participants, found it challenging to set aside clinical instincts when participants became distressed, and these experiences were explored in supervision and team discussions.

Finally, as a team we reflected on the relative homogeneity of our sample, which consisted mainly of white, middle-class mothers. Despite efforts to recruit widely, time constraints limited our ability to access a more diverse sample. We recognised this as a limitation, both within our study and more broadly in eating disorder research, and we remain conscious of whose voices continue to be underrepresented in the literature.

## Abbreviations

AN: Anorexia nervosa
ASC: Autism spectrum conditions
PEACE Pathway: Pathway for eating disorders and autism developed from clinical experience pathway
YP: Young people
IPA: Interpretative phenomenological analysis
EDS: Eating disorders service
PES: Personal experiential statements
HRA: Health research authority
FBT: Family-based therapy
